# Failure pattern and radiotherapy exploration in malignant pleural effusion non-small cell lung cancer treated with targeted therapy

**DOI:** 10.3389/fonc.2023.974735

**Published:** 2023-05-19

**Authors:** Qingsong Li, Cheng Hu, Shengfa Su, Zhu Ma, Yichao Geng, Yinxiang Hu, Huiqin Li, Bing Lu

**Affiliations:** ^1^Department of Thoracic Oncology, Affiliated Hospital of Guizhou Medical University, Guiyang, China; ^2^Department of Thoracic Oncology, Affiliated Cancer Hospital of Guizhou Medical University, Guiyang, China; ^3^Teaching and Research Department of Oncology, Clinical Medical College of Guizhou Medical University, Guiyang, China

**Keywords:** non-small cell lung cancer, malignant pleural effusion, failure pattern, radiotherapy, targeted therapy

## Abstract

**Purpose:**

Actionable mutations are common in non-small cell lung cancer(NSCLC)with malignant pleural effusion(MPE)(MPE-NSCLC). The pattern of failure in MPE-NSCLC treated with targeted therapy after MPE control remains unclear. We aimed to investigate the failure pattern of such patients in a cohort study and explore the possibility of radiotherapy.

**Patients and methods:**

Computed tomography scans of 86 patients were reviewed in this study. We classified first pattern of failure after MPE control as initial disease sites only (IF), new distant sites only (NF), or IF and NF detected simultaneously (INF). Patients evaluated suitable for radiotherapy after disease progression were divided into two groups: D group without radiotherapy and RD group with radiotherapy. The Kaplan-Meier method and log-rank test were used for survival analyses.

**Results:**

Disease progression after MPE control was observed in 42 patients with complete serial imaging. Median time to any progression was 9.5 months. Rate of the IF, NF and INF were 50%, 17% and 33% for all patients,60%,0% and 40% for patients with MPE recurrence (n=10,23.8%) and 47%, 22% and 31% for patients (n=32,76.2%) without MPE recurrence, respectively. Out of 10 patients(23.8%) with MPE recurrence, 7 patients simultaneous underwent primary tumor progression and 5 MPE were cytologically confirmed in 7 patients with examination. The overall survival (OS )rates at 1, 2, 3 years for the RD group and D group were 88.2%, 50.5%, 21.7% and 80.0%, 20.3%, 0%, respectively; the corresponding MST were 26.1 months and 17.5 months, respectively (χ2 = 4.959, p =0.026)

**Conclusions:**

Our data indicates that 50% of patients with actionable mutations MPE- NSCLC after MPE control are likely to fail at their initial sites of disease and the use of radiotherapy may bring OS benefits during the course of their disease. Multicenter RCT is necessary to confirm the result in the future.

## Introduction

Malignant pleural effusion(MPE) is a common clinical problem in patients with malignant tumors, in which lung cancer is the most common, accounting for about 37.5% (1). Lung cancer remains one of the most common cancer in the world, with approximately 2.2 million new patients diagnosed every year, and 86% of diagnosed patients have non-small cell lung cancer (NSCLC) (2), approximately 20% of patients with lung cancer develop MPE during the course of their disease (3), which is associated with a poor prognosis (4, 5) and reduce quality of life (6, 7).

Over the past decade, first-line treatment of stage IV NSCLC has evolved from chemotherapy alone to chemotherapy, targeted therapy (8–11), and immunotherapy (12, 13). Oncogene-driven cancers represent a unique subset of non–small-cell lung cancer (NSCLC) that responds well to tyrosine kinase inhibitors(TKIs). Clinically, the use of TKI in patients with EGFR-mutant(EGFR-M) NSCLC and anaplastic lymphoma kinase-positive(ALK-P) are associated with an objective response rate(ORR) of more than 70%,and disease control rate (DCR) of 90-100%, and significantly prolong progression-free survival (PFS) (14–17). However, acquired resistance inevitably occurs in most patients after 1-2 years (18, 19). Suchit H. Patel et al. found that the main pattern of progression in EGFR-M NSCLC is in the initial sites of disease (Rate, 79.6-82.4%) (20, 21). Several studies (22–26) have demonstrated that PFS and overall survival(OS)can be significantly prolonged through local therapies, such as radiotherapy.

However, all of the above studies excluded patients with MPE-NSCLC. The pattern of failure and whether radiotherapy can improve survival remain unclear in actionable mutations MPE-NSCLC treated with targeted therapy. The aim of this cohort study was to investigate the failure pattern of such patients and explore the possibility of radiotherapy.

## Materials and methods

### Patients

A retrospective study was conducted in patients with EGFR-M or ALK-P NSCLC who had MPE from January 2014 to December 2020 at Affiliated Cancer Hospital of Guizhou Medical University(the 7th edition of the American Joint Committee on Cancer staging system). We searched for potential patients in electronic medical records system by several keywords, which were targeted drugs available in our hospital (gefitinib, icotinib, erlotinib, osimertinib, crizotinib and alectinib), MPE and NSCLC. Patients who met the following criteria were enrolled: (1) histologically or cytologically confirmed MPE-NSCLC with EGFR-M or ALK-P(next-generation sequencing (NGS) used in determining molecular testing with tumor tissue or MPE or blood);(2) treated with targeted therapy without local therapy before disease progression; (3) 18 years of age or older; (4) no previous malignancy or other concomitant malignant disease;(5) tumor stage was assessed by systemic imaging(either Contrast-enhanced computed tomography [CT] of the chest, abdomen or positron-emission tomography [PET]/CT) and brain imaging (either contrast-enhanced CT or magnetic resonance imaging[MRI]); (6)Follow-up scans must include CT of the chest and abdomen (7)Follow-up scans must include bone scintigraphy or/and imaging of the brain if patients had corresponding clinical symptoms(e.g, bone pain or headache.

Baseline characteristics were obtained from electronic medical records and department of epidemiology, including age, gender, Karnofsky performance status (KPS), histology, TNM stage, actionable mutations, metastasis status, and subsequent treatment. This study was approved by the ethics committee of affiliated Hospital of guizhou medical university.

### Treatment outcome and pattern of failure analysis

Baseline imaging included computed tomography (CT) of the chest and abdomen, with or without positron emission tomography(PET)/CT. Follow-up scans included CT of the chest and abdomen at least. All serial imaging were assessed by a senior oncologist for sites of initial disease (primary and metastatic), disease response to targeted therapy, and tumor progression in initial (primary and metastatic) or new sites of tumor. Tumor response was scored according to the Response Evaluation Criteria in Solid Tumors 1.1 criteria (27). Response of MPE scored according to the Response Criteria from the Japan Lung Cancer Society (28–30) and MPE control was defined as efficacy of complete response(CR)or partial response(PR). MPE recurrence was defined as appreciable progression of effusion (at least a 20% increase in the volume of pleural effusion) (28–30). Initial disease failure (IF) was defined as evidence of the primary and individual metastatic lesions that were present before initiation of targeted therapy and had progression. New distant failure(NF) was defined as the radiographic appearance of a new metastatic lesion that was not identified at disease presentation before initiating Targeted therapy. Initial disease and new distant failure (INF) was defined by evidence of IF and NF detected simultaneously on follow-up imaging.

### Potential eligibility of radiotherapy

The CT and MRI scan at the disease progression was retrospectively assessed by a radiation oncologist in this study to determine if the extent of progression disease was hypothetically amenable to radiotherapy, including MPE control status, limited disease progression(defined as ≤ 3 organs)excepted for extensive metastases.

### Statistical analysis

All patients experienced progression before death. PFS was measured from the time of targeted therapy initiation until progression on the same targeted therapy according to RECIST 1.1 criteria. OS (31) was defined as the interval between the date of systemic therapy and the date of death due to any cause. Dichotomous variables were presented as counts and were analyzed using Pearson’s chi-square test or Fisher exact test. The Kaplan-Meier method was used for survival analysis, and the log-rank test was used for between-group analysis. All statistical tests were 2-sided, and P values <0.05 were considered statistically significant.

## Results

### Patient characteristics

**Figure 1:**
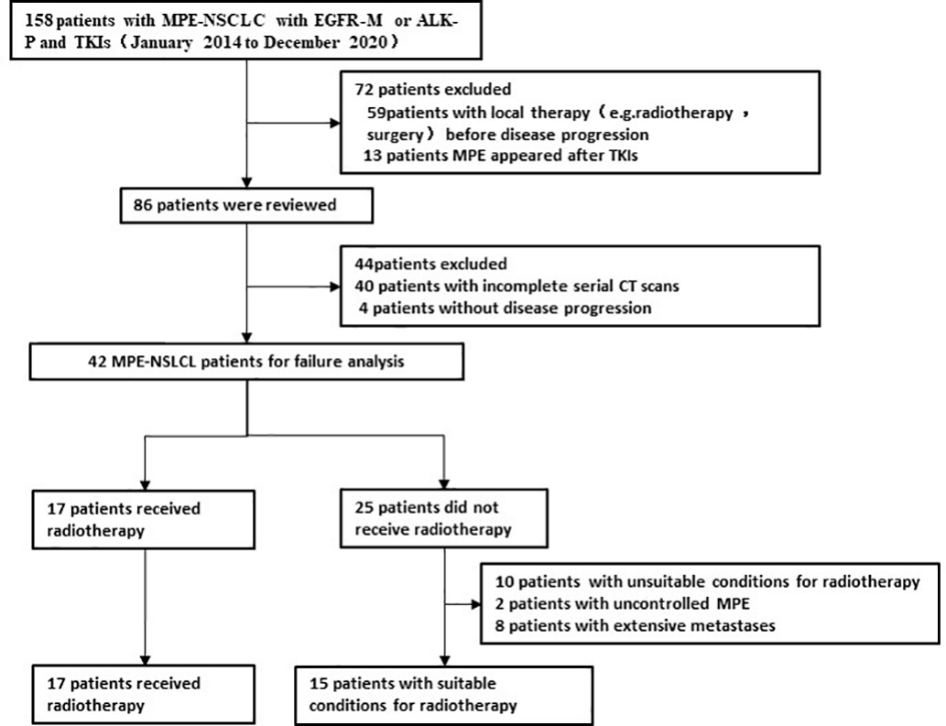
Flowchart of patient cohort.

From January 2014 to December 2020, out of 158 patients with MPE- NSCLC treated with targeted therapy, 42 patients without local radiotherapy before disease progression had complete serial CT/MRI scans for a review of the pattern of failure (**Figure 1**). There were 28 females and 14 males. The median age was 63 years (28–79 years, age ≤ 70 years in 32 patients), and the median KPS was 80. The common site of metastatic disease at initial diagnosis was the bone, lung, and brain and the majority of patients (n=25,59.5%)had involving organ in 1-3 organs (**Table 1**).

**Table 1:** Patient characteristics and failure pattern in all site.

characteristic		n=42	Disease progression		
			IF (n=21)	NF (n=7)	INF (n=14)
Gender	Male	14	6	3	5
	Female	28	15	4	9
Age (years)	<63	20	11	5	4
	≥63	22	10	2	10
T stage	T1–2	10	6	2	2
	T3–4	32	15	5	12
N stage	N0–2	19	13	4	2
	N3	23	8	3	12
Simple MPE	Yes	6	4	1	1
	No	36	17	6	13
Other metastases					
Lung	Yes	17	9	2	6
	No	25	12	5	8
Brain	Yes	12	5	2	5
	No	30	16	5	9
Bone	Yes	28	13	4	11
	No	14	8	3	3
Liver	Yes	5	3	0	2
	No	37	18	7	12
Adrenal	Yes	3	2	1	0
	No	39	19	6	14
Other	Yes	10	5	1	4
	No	32	16	6	10
Involving organ ≤ 3	Yes	25	13	5	7
	No	17	8	2	7
EGFR/ALK	EGFR	35	18	5	12
	ALK	7	3	2	2

The initial targeted therapy was administered as a first-line treatment during the course of the disease in 36 (85.7%), as a alternative treatment (KPS<70 after 1-2 cycles systemic chemotherapy) in 3(7.1%), as a second-line treatment (progressive disease after systemic chemotherapy) in 2 (4.8%), and as a maintenance therapy (partial response after 4 cycles systemic chemotherapy)in 1(2.4%). Initial targeted therapy included gefitinib(n=20), icotinib(n=12), erlotinib(n=2), osimertinib(n=1), crizotinib (n=5)and alectinib (n=2). Before MPE control, majority of patients(n=26,61.9%)had underwent indwelling pleural catheter(IPC) plus intrathoracic chemotherapy infusion[ICI; Cisplatin(80–100 mg/m2, every 21–28 days)was instilled *via* the catheter until MPE control]. Sixteen (n=16,38.1%)patients had underwent simple IPC, in which three had received IPC again due to MPE recurrence. MPE recurrence was significantly different between simple IPC and IPC plus ICI (p=0.045).

### Pattern of disease failure after MPE control

The last follow-up time was May 2022, with a median follow-up period of 19 months. All patients developed progression with a median time to progression of 9.5

months (range, 1.5–29.9 months). After MPE control, the cumulative actuarial rates of any progression at 6,12, and 18 months were 23.8%,71.4%, and 95.2%, respectively. Rate of the IF, NF and INF were 50%, 17% and 33% for all patients (n=42), 60%,0% and 40% for patients with MPE recurrence (n=10), and 47%, 22% and 31% for patients without MPE recurrence (n=32), respectively (**Table 2**). Out of 10 patients with MPE recurrence after MPE control in initial treatment, 7 simultaneously underwent primary tumor progression.

**Table 2:** Failure pattern in 42 patients with MPE-NSCLC.

Initial disease progression		All (n = 42		IF (n=21)		NF (n=7)		INF (n=14)	
		n	%	n	%	n	%	n	%
With MPE failure		10		6		0		4	
	Primary only	6	14.3	6	28.6	0	0	0	0
	Metastasis only	3	7.1	0	0	0	0	3	21.4
	Primary and metastasis	1	2.4	0	0	0	0	1	7.1
Without MPE failure		32		15		7		10	
	Primary only	8	19.0	8	38.1	0	0	0	0
	Metastasis only	15	35.7	4	19.0	7	100%	4	28.6
	Primary and metastasis	9	21.4	3	14.3	0	0	6	42.9
Sites of initial failure									
	Lung	28	66.7	18	85.7	0	0	10	71.4
	Bone	9	21.4	2	9.5	2	28.6	5	35.7
	Brain	12	28.6	1	4.8	4	57.1	7	50.0
	Other	7	16.7	0	0	3	42.9	4	28.6

### Subsequent treatment after disease failure

MPE recurrence was observed in 10 patients after MPE control in initial treatment, including 4 patients in IPC plus ICI and 6 patients in IPC(p=0.142).All patients with MPE recurrence had underwent IPC ± ICI, and 8 patients regained MPE control. Cytological confirmation was found in 5 of 7 patients with examination and bloody pleural effusion was found 1 of 3 patients without examination.

Thirty patients had received subsequent systemic treatment, including systemic chemotherapy(n=7), osimertinib(n=9), and previous targeted therapy(n=14). 17 patients had received radiotherapy for progressive site, including primary tumor (n=3), metastases(n=7), primary tumor and metastases(n=7).

### Eligibility of radiotherapy

**Figure 2:**
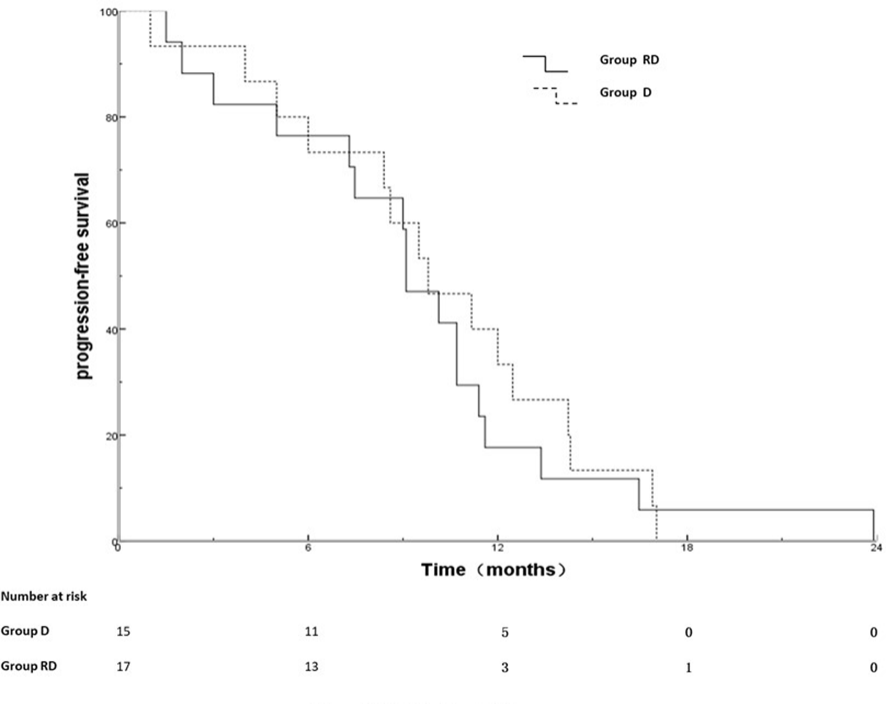
PFS for D and RD group.

**Figure 3:**
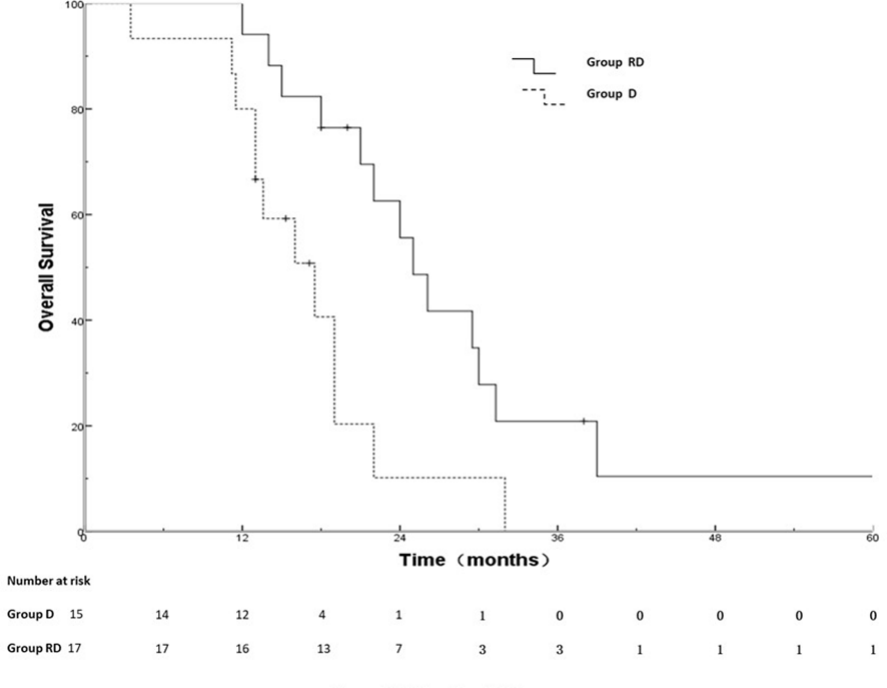
OS for D and RD group.

Out of 25 patients without radiotherapy, ten were deemed unsuitable for radiotherapy, including uncontrolled MPE (n=2), extensive progression except for MPE (n=8). Group of 17 patients with radiotherapy and 15 patients without radiotherapy was named RD and D group, respectively. There was no significant difference between the two groups with respect to gender, age, T stage, N stage, metastatic status (simple MPE vs. MPE with other metastases), involving organ (≤3 vs. >3) systemic chemotherapy and targeted therapy (**Table 3**). The PFS rates at 6, 12, 18 months for the RD group and D group were 76.5%, 17.6%, 5.9% and 73.3%, 26.7%, 0%, respectively; the corresponding median PFS were 10.0 months and 9.5 months, respectively (χ^2^ = 0.011, p =0.918) (**Figure 2**). The OS rates at 1, 2, 3 years for the RD group and D group were 88.2%, 50.5%, 21.7% and 80.0%, 20.3%, 0%, respectively; the corresponding median survival time (MST)were 26.1 months and 17.5 months, respectively (χ^2^ = 4.959, p =0.026) (**Figure 3**).

**Table 3:** Patient characteristics of RD and D group.

Characteristics		n=32	D group	RD group	χ*^2^*	*P* value
			n=15	n=17		
Gender	Male	12	7	5	1.012	0.467
	Female	20	8	12		
Age (years)	<63	16	8	8	0.125	>0.999
	≥63	16	7	9		
T stage	T1–2	7	3	4	–	>0.999
	T3–4	25	12	13		
N stage	N0–2	14	6	8	0.161	0.735
	N3	18	9	9		
Simple MPE	Yes	6	2	4	–	0.659
	No	26	13	13		
Involving organ ≤ 3	Yes	19	8	11	0.427	0.720
	No	13	7	6		
Systemic chemotherapy*(first and second-line)	Yes	12	5	7	0.209	0.726
	No	20	10	10		
Alectinib/Osimertinib(first and second-line)	Yes	10	4	6	–	0.712
	No**	22	11	11		

*first-line in 6 patients, simultaneity in 2 patients, second-line in 4 patients;

**gefitinib or icotinib or erlotinib or crizotinib; -, No value.

## Discussion

As far as we know, this is the first research specifically in actionable mutations MPE-NSCLC treated with targeted therapy to report the patterns of disease progression and analyses potential possibility of radiotherapy. Our study shows that MPE remains a major clinical challenge and IPC has fewer further pleural interventions (n=3), such as pleural drainage procedure. Our study indicates that the disease relapse occurs first in initial involved sites of disease in more 50% of MPE-NSCLC treated with targeted therapy. These findings are consistent with stage IV NSCLC without MPE in the previous studies (21, 32).

Standard treatment for MPE-NSCLC mainly aim at controlling MPE to improve the quality of life based on systematic drug treatment (33), including systemic chemotherapy, targeted therapy, and immunotherapy. Several studies have identified that EGFR-M is common in patients with MPE-NSCLC (34, 35). Yang et al (30) found that in patients with EGFR-TKI, the ORR of MPE and target lesions was 61.5% vs 88.5%, respectively. Lin et al (34) performed a prospective study with EGFR-M MPE-NSCLC patients who were treated with oral gefitinib and found the ORR rate was 92%. However, the majority of patients had underwent recurrent MPE requiring palliative intervention in these studies. Schwalk AJ (36) et al. demonstrated that patients with actionable mutations MPE-NSCLC showed a significantly higher hazard of MPE recurrence after initial thoracentesis. Another study (37) showed that out of actionable mutations MPE-NSCLC, approximately 50% experienced MPE recurrence within 30 days. Our data showed that IPC had taken effective control of MPE in the end(1 IPC in 39 patients, ≥2 IPC in 3 patients). A meta-analysis of randomized controlled trials (38) shows that IPC has fewer further pleural interventions and shorter hospital stays. In our study, MPE recurrence was significantly different between simple IPC and IPC plus ICI (p=0.045)in initial treatment and simple IPC were marginally associated with MPE recurrence after MPE control (p=0.142). It indicates that IPC plus ICI may be better treatment for MPE in initial treatment. Schwalk AJ and colleague (36) suggest that targeted therapy alone is not sufficient for managing MPE in patients with metastatic NSCLC and actionable mutations, who would benefit from a definitive management.

The mechanisms for the development of MPE is complex and not fully unclear. Proposed mechanisms for the development of MPE include local effects of the tumor, complications from treatment, systemic effects of the tumor (39), and interactions (40) between pleural-based tumor cells and the host vasculature and immune system. We found that out of 10 patients with MPE recurrence after MPE control in initial treatment, 7 simultaneous underwent primary tumor progression. Cytological confirmation was found in 5 of 7 patients with examination. The pattern of disease progression indicates that both primary tumor and MPE need to be treated and it is feasible to continue original targeted therapy (25, 41) after effective treatment (e.g. primary tumor radiotherapy, IPC plus ICI for MPE)

Acquired resistance inevitably occurs in most patients treated with TKI after 1-2 years. The median PFS in our study was 9.5 months, which was similar to the previous studies (14–17, 30). Our data show that after MPE control, the rate of the IF, NF and INF for all patients and patients with MPE continuous control were 50%, 17%, 33% and 47%, 22%, 31%, respectively. The growth of TKI-resistant clones that most often arise within sites of persistent disease (primary and metastatic) in originally involved anatomical sites (42, 43). A study performed by Al-Halabi H et al. (21) also suggest that failure in residual sites of original disease is likely to occur before the development of new sites of distant metastasis. The growth of resistant clones can lead to systemic reseeding, which results in the development of distant failures in new sites of disease in patients with prostate cancer (44) or sarcoma of soft tissue (45) or NSCLC (21). Several studies (22–26) have demonstrated that progression-free survival(PFS) and overall survival(OS)can be significantly prolonged through local therapies, such as radiotherapy. Therefore, we consider local radiotherapy may be feasible and prolong OS for patients with MPE-NSCLC(five or fewer metastases excluding primary tumor (41)) treated with targeted therapy. So far, there was only one retrospective analysis (46) suggesting that primary tumors radiotherapy may prolong OS in patients with MPE-NSCLC and should be paid more attention. So, the role of local radiotherapy in initial treatment need to be confirmed in randomized controlled trial (RCT) in the future.

Weickhardt, et al. (26) found that radiotherapy to oligoprogressive disease was associated with more than 6 months of additional disease control. A retrospective study (47) performed by Chan et al. indicated that radiotherapy may effectively extend EGFR TKI therapy for patients with oligoprogression improve PFS (3 months)and overall survival(28.2 months vs 14.7 months). So far, no clinical studies have demonstrated the value of radiation therapy after disease progression in MPE-NSCLC treated with targeted therapy. In our study, thirty-two patients were assessed by radiation oncologist as suitable for radiotherapy, of which 17 had received radiotherapy. There was no significant difference between the two groups with clinical characteristics and PFS (RD vs D group,10.0 months vs. 9.5 months). The MST of the D group in our study was 17.5 months, which was similar to the MST of 16-18 months in previous studies (34, 48, 49). Our study found that the OS rates of the RD group were significantly higher than those of the D group (MST, 26.1 months versus 17.5months,p=0.026). Our study indicates that the use of radiotherapy may bring OS benefits after disease progression for MPE-NSCLC treated with targeted therapy.

We acknowledge several limitations to the current study. First, our study included a relatively small number of patients. Second, the pattern of disease progression and OS at other institutions might differ due to a single-institution study. Finally, this was a retrospective study with some unaccounted confounders, selection bias and recall bias. Therefore, multicenter RCT is necessary to confirm the result in the future.

## Conclusions

Our data indicates that 50% of patients with actionable mutations MPE- NSCLC after MPE control are likely to fail at their initial sites of disease and the use of radiotherapy may bring OS benefits during the course of their disease. This analysis provides further support for prospective study to confirm the role of local therapy in patients with actionable mutations MPE-NSCLC, including when and how to perform. Multicenter RCT is necessary to confirm the result in the future.

## Data availability statement

The raw data supporting the conclusions of this article will be made available by the authors, without undue reservation.

## Ethics statement

The studies involving human participants were reviewed and approved by Ethics Committee of Affiliated Cancer Hospital of Guizhou Medical University, Guiyang, China. The patients/participants provided their written informed consent to participate in this study.

## Author contributions

BL designed the study. QL,CH, SS, ZM, YG, YH, and HL collected the data. BL and QL undertook the data analysis and interpretation, and wrote the report. BL and QL carried out the statistical analysis. All authors contributed to the article and approved the submitted version.
